# Illness Uncertainty and Coping Strategies Among Families of Children with Cancer in China: A Family-Centered Qualitative Study

**DOI:** 10.3390/healthcare14142127

**Published:** 2026-07-15

**Authors:** Hui Hou, Tian-Ming Zhang

**Affiliations:** 1 Department of Social Work, Hangzhou Normal University, Hangzhou 311121, China; 20190054@hznu.edu.cn; 2 Department of Social Work, Shanghai University, Shanghai 200444, China

**Keywords:** pediatric cancer, illness uncertainty, coping strategies, family-centered care

## Abstract

**Background/Objectives:** Illness uncertainty is a pervasive psychosocial experience in chronic conditions that is particularly prominent in pediatric oncology. While existing research has explored its psychological impact, a gap remains in understanding how this uncertainty evolves throughout the disease trajectory and how families collectively negotiate and manage this experience over the long term. **Methods:** This qualitative study was conducted in the hematology ward at a pediatric hospital in Shanghai, China. Using purposive sampling, semi-structured interviews were performed with 32 participants from 12 families of children currently undergoing cancer treatment. Data were collected through in-depth interviews and analyzed using reflexive thematic analysis. The sample was dominated by leukemia cases, with a small number of lymphoma cases; therefore, the findings are most directly transferable to families of children with hematological malignancies. **Results:** Illness uncertainty is a dynamic and persistent experience permeating the entire pediatric cancer trajectory. Key sources of uncertainty include diagnostic ambiguity and delays, barriers in physician–patient communication, and profound disruptions to family daily life. In response, families proactively develop multidimensional coping strategies: reframing meaning to accept uncertainty, reorganizing family roles and responsibilities, strengthening internal communication, and mobilizing external support networks. These strategies demonstrate both family resilience and inherent vulnerability under sustained pressure. **Conclusions:** Illness uncertainty in pediatric cancer transcends medical boundaries and is deeply embedded in family life. Healthcare systems should recognize uncertainty as a core experience throughout the disease process and provide family-centered psychosocial and structural support. Strengthening hospital social work services and fostering synergy between peer networks and community resources are essential to enhancing families’ capacity to manage uncertainty and alleviating their long-term psychosocial burden.

## 1. Introduction

Illness uncertainty is widely regarded as a core psychosocial experience within the context of chronic illness. It refers to a state in which individuals, due to insufficient information or ambiguous cues, are unable to comprehend the meaning of illness-related events or to predict their outcomes [1]. This experience may persist across different stages of treatment [2]. With advances in medical care prolonging survival, an increasing number of conditions once framed primarily in terms of cure or death have been transformed into chronic states requiring long-term management. Accordingly, uncertainty has shifted from a transient experience to an enduring condition of everyday life. This shift calls for closer attention to how individuals and families experience and manage uncertainty over time.

Over recent decades, research on illness uncertainty has developed primarily around three interrelated areas: its sources, consequences, and coping processes. Existing studies consistently identify ambiguity in illness status, insufficient information, treatment complexity, and the unpredictability of prognosis as the principal sources of uncertainty [3,4,5,6,7]. Beyond its origins, a growing body of evidence has shown that persistent uncertainty contributes to psychological distress, reduces quality of life, complicates treatment decision-making, and challenges long-term adaptation for both patients and their families [2,5,6]. In response, two complementary perspectives on coping have emerged. One seeks to reduce uncertainty through improved communication, patient education, and psychosocial interventions [8]. The other focuses on managing uncertainty, framing uncertainty not as a condition to be eliminated but as an integral part of living with chronic illness [1,7,9]. Taken together, existing research has established illness uncertainty as a psychosocial process, but less is known about how it is experienced and managed in specific illness contexts where treatment trajectories and family life are deeply intertwined.

Within pediatric oncology, illness uncertainty is particularly pervasive because childhood cancer is characterized by prolonged treatment, fluctuating symptoms, uncertain prognoses, and repeated transitions between hospital and home [10,11,12,13,14]. This experience has been vividly described as “nothing is carved in stone” or “living with a time bomb” [15,16]. Empirical studies indicate that sustained uncertainty significantly intensifies psychological distress among families of children with cancer [17], increases levels of parental trait anxiety and depression [18], and negatively affects both children’s treatment outcomes and overall family quality of life [19,20,21]. To cope with these challenges, families attempt to respond by maintaining hope, revising life plans, renegotiating family roles, sustaining communication within the family, and seeking support [22,23,24,25]. Overall, this body of research has shown that uncertainty in pediatric oncology is not only persistent across the treatment process, but also closely linked to family distress, adaptation, and coping.

Recent pediatric oncology literature has further clarified that uncertainty is not a single emotional response to diagnosis but a multidimensional and time-sensitive process. A recent systematic review shows that parental uncertainty in childhood cancer is shaped by illness-related ambiguity, insufficient information, perceived disease severity, communication with clinicians, and available support, and is associated with psychological distress, decision-making difficulties, and quality-of-life concerns [26]. Evidence on family functioning also indicates that family cohesion, expressiveness, and support are associated with child and sibling adjustment following pediatric cancer diagnosis [27]. These findings suggest that illness uncertainty should be examined not only as an individual psychological state but also as a relational process embedded in family interaction.

Nevertheless, although research on uncertainty in pediatric oncology has expanded in recent years, the literature remains relatively limited in several important respects. First, studies that directly examine illness uncertainty from a whole-family perspective are still scarce, with most research focusing on parents rather than on how children, mothers, and fathers jointly experience and negotiate uncertainty. Second, uncertainty has often been examined at particular moments of diagnosis, treatment, or survivorship, rather than as a process that evolves across the entire illness trajectory. Third, while communication, support, and family functioning have been identified as relevant factors, relatively few studies have explored how these relational processes shape families’ everyday coping with uncertainty over time. Addressing these gaps, the present study adopts a whole-family perspective to explore the sources of illness uncertainty and coping strategies among families of children with cancer. By examining how uncertainty unfolds across the illness trajectory and how family members experience and cope with uncertainty in relational contexts, this study seeks to provide empirical evidence for the development of family-centered supportive care.

## 2. Materials and Methods

### 2.1. Study Design

This study employs a qualitative research design to explore how families of children with cancer perceive, experience, and construct meaning around uncertainty, and to capture aspects of everyday life that are difficult to access through quantitative approaches [28]. The study was underpinned by critical realism as its philosophical foundation. Critical realism views objective realities and subjective interpretations not as opposing categories, but as dynamically intertwined [29]. From this perspective, we assumed that uncertainty is rooted in the realities of illness and treatment, while families interpret and respond to it within broader relational, cultural, and structural contexts. This position enabled the study not only to explore what uncertainties families encountered, but also to examine how they made sense of and navigated these uncertainties in everyday life. Reporting follows the Consolidated Criteria for Reporting Qualitative Research (COREQ, see Supplementary Materials File S1).

### 2.2. Sample and Recruitment

The study population consisted of families of children with cancer. Participants were recruited via purposive sampling based on the following inclusion criteria: (1) families with a child diagnosed with pediatric cancer and currently undergoing active treatment; and (2) both the child and their family caregivers possessed the cognitive and communicative capacity to participate and provided voluntary consent.

Recruitment took place in the hematology ward of a pediatric hospital in Shanghai, China. From September 2017 to February 2019, the first author conducted field observations in the ward and invited eligible families through open recruitment. Written informed consent was obtained from the family caregivers of each child participant after they had received a comprehensive explanation of the study objectives, interview procedures, confidentiality protections, and the voluntary nature of participation. Children who were able to understand the study were provided with age-appropriate explanations and were invited to provide written or pictorial assent prior to the interview. Both caregivers and children were informed that they could decline to answer any question or withdraw from the interview at any time without consequence. Interviews were conducted at locations convenient for the participants, primarily in the hospital’s social work office or the families’ homes. Data were collected and analyzed concurrently. Following each round of interviews, the research team reviewed interview logs, field notes, and preliminary coding to assess the richness, diversity, and adequacy of the data in addressing the study aims. Particular attention was paid to whether newly recruited families contributed substantively new insights into the sources of uncertainty, families’ interpretations of uncertainty, and their coping strategies, or extended the developing analytic framework. After the 12th family was included, subsequent interviews primarily elaborated and refined existing themes without generating new conceptual insights or expanding the analytic framework. The research team therefore determined that sufficient depth and breadth of data had been achieved and ceased recruitment.

### 2.3. Data Collection

Data were collected by the first author through semi-structured interviews, all of which were audio-recorded with the participants’ consent. To ensure that each family member could express their views independently, interviews were conducted separately whenever possible. Only two children (F02 and F04) were accompanied by a parent due to their physical condition. For younger children, interviews tended to be shorter, used simpler and more concrete language, and drew upon children’s everyday experiences to facilitate expression. For example, broad questions such as “How has the illness affected your daily life?” were rephrased into more concrete prompts, such as asking what a typical day in the hospital was like, what they usually did when they felt bored or worried, and who they would turn to for help. The interviewer remained attentive to children’s verbal and non-verbal cues and adjusted the pace and depth of questioning according to the child’s comfort, engagement, and capacity to participate. A total of 32 interviews were completed, with each interview lasting between 20 min and 2 h.

The interview guide was jointly developed by the research team and finalized following team discussion and pilot interviews (see Supplementary File S2). Through open-ended questions, participants were invited to reflect on their experiences since the child’s illness began, with the interviews gradually focusing on experiences of illness uncertainty and related coping processes.

### 2.4. Data Analysis

Data were analyzed by reflexive thematic analysis, as developed by Braun and Clarke [30]. This method was chosen because of its flexibility and applicability across different philosophical frameworks [31]. The analysis proceeded through six phases.

First, recordings were transcribed verbatim, and the transcripts were read repeatedly to achieve familiarity with the data. Reflective notes, initial observations, and preliminary analytic ideas were recorded during this phase. Second, initial coding was conducted using an inductive approach, identifying significant concepts emerging from the data. A single code was sometimes relevant to more than one theme. Third, the initial codes were grouped into potential themes. Similar codes were reviewed and categorized, and the interview data were checked to ensure that the themes were supported by the participants’ accounts. Relevant themes were discussed collaboratively by the research team. Fourth, following these discussions, the dataset was again reviewed to further revise and refine the themes. Some overlapping themes were merged, and themes supported by too few codes were removed. Fifth, the themes and subthemes were further defined, refined, and named to ensure that they were internally coherent, logically consistent, and relevant to the research questions, and the relationships among them were clarified through detailed discussion. Sixth, the first author drafted the research report. Throughout the analytic process, the three-member research team, which included two doctoral students in social work and one faculty member in social work, held regular meetings to critically review coding decisions, challenge emerging interpretations, discuss analytic progress, and refine the developing themes, thereby enhancing the credibility and rigor of the findings.

### 2.5. Researcher Reflexivity

Reflexivity was embedded throughout the research process and regarded as a shared responsibility of the research team. At the time of the study, the first author was a female doctoral student in social work with training in medical social work and a research interest in the psychosocial well-being of children with chronic illnesses and their families. Prior to the study, she had completed an internship as a medical social worker in the participating hospital, facilitating activities for hospitalized children and their families. While this experience enhanced contextual understanding and facilitated rapport with participants, it also carried the potential risk of shaping assumptions about participants’ experiences.

To address these influences, the first author maintained reflexive interview logs and field notes throughout data collection and analysis, documenting observations, emotional responses, emerging interpretations, methodological decisions, and reflections on interviewing practices. Particular attention was paid to how prior professional experiences might influence what was attended to, emphasized, or overlooked during the research process. The first author also consciously avoided reverting to a service-provider role and sought to allow participants to guide discussions toward issues they considered meaningful.

The second author, also a doctoral student in social work, and faculty members with expertise in social work research participated in regular analytic discussions. These discussions critically examined interview strategies, the adequacy and richness of the data, emerging interpretations, and judgments regarding data saturation. Through ongoing reflexive dialogue, the research team sought to challenge unexamined assumptions and enhance the credibility and rigor of the analytic process. Reflexivity was therefore treated not as a discrete methodological step, but as an ongoing practice shaping data generation, interpretation, and representation.

## 3. Results

A total of 32 participants from 12 families were interviewed, consisting of 12 children with cancer and 20 adult family members. The ages of the children ranged from 4 to 13 years, while the ages of the family members ranged from 28 to 46 years. The participating family members included 12 mothers and 8 fathers; the remaining 4 fathers were unable to participate due to work commitments in other locations. The sociodemographic characteristics of the sample are presented in Table 1.

**Figure 1:**
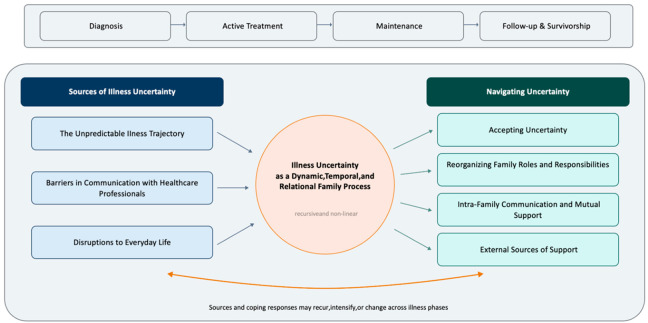
Dynamic relationships between sources of illness uncertainty and family coping strategies across the pediatric cancer trajectory.

The thematic analysis generated two main themes and seven subthemes, as shown in Table 2. The dynamic relationship between the sources of illness uncertainty and families’ coping strategies across the pediatric cancer trajectory is summarized in Figure 1. The figure illustrates how different sources of uncertainty may recur and change over time, while families continually adjust their coping responses across diagnosis, active treatment, maintenance, follow-up, and survivorship.

### 3.1. Being Uncertain: Sources of Uncertainty Among Families of Children with Cancer

#### 3.1.1. The Unpredictable Illness Trajectory

Most participants experienced uncertainty arising from the ambiguity and delay surrounding the diagnostic period. Notably, all 12 families went through 2 to 5 referrals, with the diagnostic process lasting from 10 to 180 days. During the repeated medical consultations and the prolonged wait for a definitive diagnosis, families frequently speculated about the severity of the illness and faced considerable uncertainty in deciding which hospital to attend and which doctor to consult. As F04m explained: *We didn’t know which hospital to go to or which doctor to see. We could only search online blindly, and we were terrified that choosing the wrong one would delay the child’s treatment.* Some families, such as F02, also experienced misdiagnosis or ineffective treatment. The combination of delayed diagnosis and growing distrust in the healthcare system further intensified their sense of uncertainty.

*At first, my child was diagnosed with a cold. Later, when the doctors still could not figure out what was wrong, they even suspected it was a psychological problem related to school refusal. It was only after 95 days that my child was finally diagnosed with leukemia. During all that time, my child’s condition kept getting worse at home, and we became more and more afraid that the hospital and the doctors had delayed the diagnosis and treatment.*

(F02f)

Once treatment began, uncertainty mainly stemmed from the ongoing fluctuations in treatment effects and physical reactions. The timing and severity of chemotherapy side effects and complications were difficult to predict, rendering previous experience unreliable and often necessitating repeated adjustments to treatment plans. This, in turn, heightened families’ uncertainty about the treatment process. As F04m shared: *The hardest thing to predict is the side effects. They are different every time, and that is really frightening.* Such individual variability renders prior experience difficult to replicate or draw upon, leaving families to confront a new situation of uncertainty with each course of treatment. As F04c stated: *I am very afraid of chemotherapy drugs. Once they are infused, it feels like a ticking time bomb; you never know what they might bring.* Taken together, the accounts of F04m and F04c suggest that uncertainty surrounding treatment was shared within the family but experienced differently by its members. While the mother emphasized the unpredictability of treatment effects, the child focused on the immediate fear associated with chemotherapy and its unknown consequences. These converging yet distinct perspectives illustrate how uncertainty was collectively experienced within families, even as its meanings and expressions varied across family members.

In the later stages of treatment and during the maintenance phase, uncertainty was primarily centered on concerns about recurrence and whether the child would be able to return to a normal life. While gradually attempting to restore a sense of normality, families remained highly sensitive to fluctuations in follow-up test results and physical indicators. F05m compared this experience to *a sword hanging over our heads.*

#### 3.1.2. Barriers in Communication with Healthcare Professionals

Limited comprehensibility of medical information was another major source of uncertainty for families. During diagnosis and medical decision-making, physicians often communicated using professional terminology or probabilistic expressions. Given their limited medical knowledge, families frequently struggled to understand this information and, as a result, translated uncertainty into fears of the worst possible outcome. As F03m stated: *The doctor used so many medical terms that we basically couldn’t understand anything. We just felt it must be an incurable disease, which was terrifying.* This uncertainty did not remain an individual reaction but entered subsequent family discussions, where it was collectively interpreted and sometimes intensified. As F03f noted: *The more my wife and I discussed it, the more frightened we became*.

In contexts where healthcare professionals had limited time and provided insufficient emotional support, families were not only left to process medical uncertainty on their own but also expected to bear responsibility for treatment-related decisions. As F07m described: *The doctor said there was a strong suspicion of leukemia, but how serious it was could only be determined once treatment began. They also said that even medicine could not fully explain it. Hearing that was frightening. Before you even had time to process it, they moved straight on to discussing the next treatment plan.* The doctor’s comments further amplified the family’s experience of uncertainty, as they were suddenly confronted with a highly threatening but not yet fully explainable condition. Moreover, the rapid transition from diagnostic suspicion to treatment planning left them with little time to process the news, make sense of the illness, or regain a sense of control. Under these circumstances, ambiguous medical communication quickly became a matter for family discussion and decision-making. Family members had to interpret incomplete information together, weigh possible consequences, and make treatment-related decisions without clear answers. In this sense, communication barriers did not only create individual anxiety but also transformed uncertainty into a collective family burden.

#### 3.1.3. Disruptions to Everyday Life

The illness profoundly disrupted the spatial and temporal organization of family life, introducing a major source of uncertainty. Frequent hospitalizations, follow-up visits, and temporary relocation for treatment removed families from their previous routines and reference points. Everyday life was reorganized around treatment-related activities such as intravenous infusions and medication schedules. As F10f described: *Sometimes I don’t even know what day of the week it is. I only remember how many days we’ve been in the hospital.* This suggests that the family’s sense of time had shifted from socially structured time to treatment-oriented time, with the rhythm of daily life being completely reconfigured. Family travel, leisure, and entertainment were all forced to give way to medical arrangements. As F09m noted: *Life can no longer be described as normal. We used to travel and have family gatherings, but now we do not dare to do those things for fear that something might happen to the child.* Given the prolonged treatment trajectory, families remained uncertain about when, or whether, life would return to “normal,” thereby generating a persistent sense of long-term uncertainty.

Prolonged medical residence and caregiving demands also weakened families’ connections with their work, school, and community networks, reducing their access to external information and emotional support. As F10m described: *Since my child became ill, I have basically had no social life. I am no longer working, nor am I in my original living environment—it feels like being pulled out of the soil like a radish.* The intensive demands of caregiving deprived parents of their everyday social interactions and usual sources of emotional support, while the child was also separated from school and their peer network. As a result, families were often left to absorb and manage uncertainty largely within the family itself.

In addition, the long-term and fluctuating costs of treatment, coupled with ongoing caregiving demands and the frequent need for primary caregivers to resign or reduce their income, often placed families in a struggle between sustaining care and maintaining their livelihood. In this way, uncertainty extended beyond the medical sphere and became an enduring experience embedded in the family’s overall living conditions.

### 3.2. Navigating Uncertainty

#### 3.2.1. Accepting Uncertainty

When confronted with prolonged and difficult-to-eliminate uncertainty, families of children with cancer gradually developed a meaning-making orientation centered on acceptance, incorporating uncertainty into their life narratives. Some families reinterpreted the illness experience as an opportunity to reassess life and their priorities. As F12m explained: *Isn’t the child’s illness also an opportunity for change? It has made us realize that health is what matters most.*

Some families drew on Chinese cultural beliefs, such as the idea that fortune and misfortune coexist, to place the illness within an understandable interpretive framework.

*There is an old saying that fortune and misfortune come together. So, I tell myself that after this illness, maybe greater blessings are still waiting for my child.*

(F07f)

In this way, families were able to assign positive meaning to uncertainty and attain some psychological peace. Other families reconstructed uncertainty as a state in which hope still remained by comparing the current situation with worse possible outcomes. As F03m stated: *Doesn’t uncertainty also mean there is still a chance? If the doctor had immediately given us a death sentence, that would have been the real end.* Overall, rather than remaining trapped in uncertainty, families gradually came to accept it through processes of meaning reconstruction.

#### 3.2.2. Reorganizing Family Roles and Responsibilities

The child’s illness disrupted the family’s established mode of functioning, and most families responded to uncertainty by reorganizing the division of labor within the household. A common arrangement involved the mother leaving employment to care for the child, while the father continued working to maintain the family income. However, the arrangements surrounding these roles were continually adjusted as families sought to balance caregiving needs, financial responsibilities, and the changing demands of treatment. As F06f explained: *After discussing it with my wife, we decided that she would stay with our child while I kept working. But as the treatment demands increased, we had to keep adjusting our arrangements. Eventually, I changed jobs so that I could take on more caregiving responsibilities whenever I was needed.*

Some fathers, such as F10f and F11f, were able to achieve a balance between work and caregiving because they held relatively stable or institutionally protected jobs, allowing them to use flexible leave, work online, or take short absences. In contrast, fathers engaged in labor-intensive or informal employment, such as F02f, F03f and F06f, were often forced to quit their jobs and turn to flexible forms of employment, such as food delivery or designated driving services, to gain greater control over their time. This flexibility, however, came at the cost of physical exhaustion and long-term instability. As F02f noted: *During the day, I have to help take care of the child, and at night, I do designated driving. I definitely won’t be able to keep this up in the long run.* These accounts suggest that while flexible employment may alleviate short-term difficulties, it is unlikely to serve as a sustainable long-term coping strategy.

At the same time, some mothers attempted to take on online part-time work once the child’s condition had become relatively stable. This not only helped ease the financial burden of families but also enabled them to regain a sense of control over life and a sense of self-worth. For some mothers, returning to work also represented an effort to renegotiate their roles within the family beyond the identity of caregiver. For example, F07m and F09m ran online micro-businesses, while F08m shifted to online insurance sales.

*This online job keeps me from constantly dwelling on my child’s illness. I feel less anxious, and earning money gives me a real sense of accomplishment. It feels like one of the few things I can still control.*

(F07m)

*Although the income is not much, it still helps support the family. When I have time while caring for my child, I post on WeChat Moments. I am very satisfied with this job.*

(F09m)

Overall, the reorganization of family roles was not a one-time adjustment but an ongoing family process through which members sought to sustain everyday functioning under conditions of uncertainty. Although these adaptive arrangements enhanced families’ coping capacity to some extent, they also redistributed burdens and generated new vulnerabilities within the household.

#### 3.2.3. Intra-Family Communication and Mutual Support

In the face of uncertainty, many families engaged in more frequent and open communication within the household. Communication within the family provided an important space for members to make sense of uncertainty together, share emotional burdens, and coordinate their responses to ongoing challenges. Emotional disclosure between spouses became an important mechanism for emotional regulation. For example, F10f would listen to his wife express her frustrations and worries related to various uncertainties: *Sometimes, all the uncertainty makes my wife really anxious. I just let her talk and vent, so she can get those negative feelings off her chest*. F10m said: *After the child became ill, his father and I talked a lot. It felt like we were facing everything together.* This shared sense of confronting the illness together helped transform individual emotional distress into collective family coping. The paired accounts from F10 show how uncertainty was negotiated within the marital relationship rather than managed by each parent separately. While the father described his role as listening and containing his wife’s anxiety, the mother emphasized the sense of jointly facing the illness through repeated conversations. Their perspectives converged around the need to face uncertainty together, while also revealing different emotional roles within the family. In this way, communication transformed individual distress into a shared family process of emotional regulation and collective coping.

At the same time, some families also attempted to communicate more openly with the child about the illness, helping them understand the goals of treatment and participate in self-management. For example, F01m initially avoided discussing the illness out of concern for her child’s emotions, but this led to poor treatment adherence. However, after attempting more open and honest communication, the child became more cooperative. As F01m noted: *I did not expect that after our conversation, she really would begin to take eating seriously, which also reassured us a great deal.* This suggests that open and honest communication not only facilitates the child’s health management but also strengthens trust and shared understanding within the family. Through this ongoing interaction, parent–child relationships shifted from one-way caregiving toward mutual understanding and emotional support. As F10m explained: *I also talk to my child about my fears, and she comforts me. Suddenly, it feels good that we understand each other, and I’m not so afraid anymore.*

#### 3.2.4. External Sources of Support

In addition to drawing on internal family resources, families actively mobilized external support networks to cope with uncertainty. Relatives, particularly grandparents, provided crucial assistance both financially and in caregiving. For example, elders in F08 contributed their savings, while those in F12 took turns caring for the child, cooking meals, and delivering food. These forms of support helped reduce financial and caregiving-related uncertainty.

*My mother used all her retirement savings because she was afraid we would delay the child’s treatment if we ran out of money. She also kept trying to comfort me because she worried that I was under too much emotional strain.*

(F08m)

*My mother helps prepare nutritious meals for the child at home, and my father brings them to the hospital. Sometimes, when we are too overwhelmed to manage everything, they also come to help care for the child. They have been a tremendous help to us.*

(F12m)

Extended family members and friends also offered additional support in the form of information, fundraising, and emotional encouragement. For example, relatives of F01 took the initiative to help contact doctors, while friends and family of F07 helped circulate fundraising appeals for treatment costs.

*Our relatives helped us get in touch with doctors at the hospital, search for information on Weibo and Zhihu [a Q&A platform] and assist us in making sense of the information and decisions.*

(F01m)

*At that time, we launched a fundraising campaign on Qingsongchou [a fundraising platform], and many family members and friends actively shared it on WeChat Moments. Donations from many strangers came in very quickly, and the help was tremendous.*

(F07f)

Peer families constituted another important source of support. Through online peer groups and interactions in hospital wards, families gained experiential knowledge, emotional comfort, and practical mutual aid, particularly in situations such as shortages of medication. As F04m recalled: *There was an urgent shortage of mercaptopurine, so I asked for help in the online parent support group, and many people reached out and offered us medicine.* These reciprocal support relationships reduced families’ sense of isolation amid the uncertainty and helped transform uncertainty from a private family burden into a shared experience understood by others facing similar circumstances.

A few families also received professional support from hospital social work departments. Through institutional resource linkage and emotional accompaniment, such services helped alleviate both financial and psychological stress. For example, while the child in F01 was being treated in the intensive care unit, the hospital social work department helped the family apply for foundation funding and provided emotional support and comfort. Overall, multilayered support networks formed by relatives, peers, and professional institutions served as important buffering mechanisms for families living under intense uncertainty.

## 4. Discussion

This study shows that uncertainty among families of children with cancer is not a stage-specific event; rather, it is a dynamic experience that persists throughout the illness trajectory and is continuously reconstructed over time. Uncertainty primarily arose from the limitations of diagnosis and treatment, insufficient communication between healthcare professionals and families, and the profound disruption that the illness brought to everyday family life. In response, families did not passively endure uncertainty. Instead, through ongoing interaction, they developed a set of multifaceted coping strategies, including accepting uncertainty, reorganizing family roles and responsibilities, strengthening communication with relatives, and mobilizing multilayered support networks. These findings extend existing pediatric oncology literature by showing that uncertainty is not only an individual psychological state but also a temporal and relational family process. The accounts of children, mothers, and fathers converged around shared concerns about treatment unpredictability and recurrence, while also revealing differences in how uncertainty was expressed and managed within family roles.

This family-level interpretation is important because prior research has often focused on parents as individual caregivers. A recent systematic review identifies multiple determinants and consequences of parental uncertainty in childhood cancer, while family-functioning research shows that family cohesion, expressiveness, and support are associated with better adjustment among children and siblings after a pediatric cancer diagnosis [27]. Our findings build on this evidence by illustrating how uncertainty is negotiated through everyday family practices, including role redistribution, emotional co-regulation, parent-child communication, and the mobilization of kinship and peer networks.

The emergence of uncertainty was not determined solely by the illness itself. Consistent with previous research, once treatment begins, the highly individualized nature of treatment responses and side effects often renders prior experience unreliable [32,33,34], thereby weakening families’ sense of control over the treatment process. Notably, however, uncertainty does not disappear after the completion of acute treatment. Instead, during the maintenance and follow-up phases, it becomes transformed into long-term concern about the risk of recurrence [35,36], highlighting the importance of incorporating temporality into the analytical framework [37]. This trajectory-based pattern is globally relevant, as families in different pediatric oncology settings must repeatedly reinterpret uncertain information as the child moves from diagnosis to active treatment, maintenance, follow-up, and survivorship [2,17]. Rather than being confined to a single clinical moment, uncertainty is continually reshaped by changing treatment demands, fluctuating prognostic expectations, and the family’s evolving understanding of the child’s condition.

Building on these trajectory-related findings, our study further shows that uncertainty is also shaped by healthcare communication, particularly how clinicians explain uncertain disease status, treatment effects, prognosis, and emotionally difficult information to children and caregivers [38,39]. We found that uncertainty arose not only from the complexity of medical knowledge [40], but also from the ways in which information was communicated, often with insufficient regard for families’ capacity to understand and their emotional needs. Although the use of technical terminology and probabilistic expressions may reflect professional rigor, it can also undermine families’ sense of agency [41]. Such language may be interpreted by families as signaling heightened risk, thereby intensifying uncertainty. Consistent with previous studies, the comprehensibility of medical information appears to be a key factor shaping the degree of uncertainty experienced by families [38]. This finding suggests that patient-centered and family-centered communication should encompass not only information transmission but also emotional support [39], and that techniques such as encouraging questions and engaging in hypothetical dialogue may help alleviate uncertainty [38,42].

Beyond clinical encounters, uncertainty also became embedded in the everyday organization of family life. Long-term hospitalization and frequent follow-up visit reorganized family routines around medical schedules, forcing interruptions to work, schooling, and social activities. The disruption of social networks reduced families’ access to information and emotional support, thereby exacerbating their uncertainty [43]. At the same time, the withdrawal of primary caregivers from the labor market, together with the long-term and unpredictable costs of treatment, placed families in an ongoing struggle between caregiving and livelihood [44,45,46]. As a result, uncertainty extended from the medical domain into the family’s broader living conditions. This finding aligns with international evidence that childhood cancer can have substantial socioeconomic consequences for parents and families [47].

While the mechanisms above have international relevance, several findings should be interpreted in relation to China’s healthcare system and family-cultural context. Delayed diagnosis and repeated referrals were common among the families interviewed, reflecting broader problems related to the uneven spatial distribution of medical resources and the limited diagnostic capacity of primary-level healthcare institutions [48]. Previous studies have shown that medical resources in China are highly concentrated in first-tier cities [49]. Given the underdeveloped referral system [50], families from other regions must often assume greater responsibility for information gathering and decision-making in the absence of authoritative guidance [51], while limited digital health literacy further deepens their uncertainty [52]. Recent evidence from China also shows that online health information-seeking among caregivers may shape pediatric cancer delays by influencing symptom appraisal and acceptance of clinical decisions [53]. These findings help contextualize why families in this study often relied on online searching, peer networks, and informal referrals when navigating uncertainty before and after diagnosis. The family-cultural context is equally important. In this study, uncertainty was managed not only by the nuclear family but also through broader kinship networks. Grandparents, relatives, and friends provided caregiving, financial assistance, information, and emotional support. These practices reflect the continuing influence of family-centered responsibility and intergenerational support in Chinese caregiving contexts. At the same time, reliance on family support should not be idealized, because when formal resources are insufficient, family networks may help buffer uncertainty while also redistributing burdens across generations and intensifying hidden emotional and financial strain.

Regarding coping strategies, one of the most important findings of this study is that the families commonly came to accept uncertainty through meaning reconstruction. They drew on traditional cultural beliefs, religious faith, or comparisons with prior adversity to assign understandable meaning to the illness, thereby integrating uncertainty into their life narratives. Unlike interpretations in previous studies that have treated fatalism as a negative coping strategy [54], the positive meaning reconstruction observed in this study is more consistent with Mishel’s notion of the reconceptualization of uncertainty [5] than with passive fatalistic acceptance. This finding points to the active adjustment families undertake within structural constraints [55,56,57]. Within the Chinese cultural context, expressions such as the coexistence of fortune and misfortune may function as culturally available resources for sustaining hope and relational responsibility under conditions that cannot be fully controlled.

Families also responded to uncertainty through ongoing reorganization. Consistent with previous research [58,59], the gendered pattern of mothers assuming primary caregiving responsibilities and fathers maintaining the family income remained dominant. However, this division of labor was not fixed; rather, it was continually adjusted according to treatment stage, caregiving intensity, and financial pressure. Fathers’ ability to maintain a stable income through flexible work arrangements depended heavily on their workplace conditions [60]. Although flexible employment enhanced family adaptability in the short term, it was also accompanied by reduced economic security and increased health risks [61,62]. Some mothers of children with cancer received income and a sense of self-worth through online part-time work or other forms of flexible employment, demonstrating the strategic balancing efforts families made under constrained circumstances [63].

Stronger communication within the family emerged as an important mechanism for buffering uncertainty. Consistent with previous findings [64], we found that more frequent emotional communication between spouses helped transform individual anxiety into a shared family experience of “facing it together.” The study also confirmed that more open parent–child communication promoted children’s understanding of the illness and its treatment, thereby enhancing their sense of involvement and security [65]. Together, these interactional processes strengthened the family’s adaptive capacity as a whole [66]. For practice, this suggests that psychosocial interventions should not focus only on reducing parental distress at the individual level. Instead, clinicians and social workers should assess how different family members understand uncertainty, what information is shared or withheld within the family, and how caregiving roles are negotiated over time.

Finally, consistent with previous studies [67,68], this research supports the critical role of external support networks in helping families cope with uncertainty. Grandparents, relatives, friends, peer families, and hospital social work departments provided complementary forms of financial assistance, information sharing, and emotional support, thereby serving as important buffers for families living with uncertainty. These findings not only reaffirm the function of informal support networks in illness contexts [69,70,71], but also highlight the structural potential of social work in supporting families facing high levels of risk [72]. Hospital social workers can play a central role in family assessment, resource linkage, psychoeducation, emotional accompaniment, peer-support facilitation, school re-entry coordination, and continuity of psychosocial care. International psychosocial standards emphasize that children with cancer and their families should receive systematic assessment and support from diagnosis through survivorship or bereavement [73]. Evidence from pediatric oncology social workers further shows that social work services are central to implementing these standards, although institutional resources and staffing often remain uneven [74]. In the Chinese context, strengthening hospital social work and connecting hospital-based services with community and charitable resources may reduce the extent to which families must manage uncertainty alone.

### Limitations

Several limitations of this study should be noted. First, the study was conducted in the hematology ward of a single pediatric hospital in Shanghai, and the sample size was relatively small. The participating families were dominated by leukemia cases, with only a small number of lymphoma cases. Therefore, the findings may be most transferable to families of children with hematological malignancies and should not be assumed to represent all families affected by pediatric cancer, particularly those caring for children with solid tumors or receiving care in other regions or institutional settings. Second, the study focused on children and family caregivers and did not systematically include the perspectives of healthcare professionals, such as physicians, nurses, or hospital social workers. This may have limited our understanding of how uncertainty is produced, communicated, and managed within clinical encounters and institutional systems. Third, the interviews were conducted between September 2017 and February 2019. Since then, pediatric oncology care, digital health information access, charitable support platforms, and hospital social work services in China may have continued to develop. Recent studies on online health information-seeking and financial toxicity among Chinese pediatric cancer caregivers suggest that families’ access to information, financial resources, and psychosocial support may have changed in important ways [53,75]. These developments may affect the transferability of the findings to the present context. Nevertheless, the core experiences identified in this study-diagnostic uncertainty, treatment-related unpredictability, communication challenges, disruption of everyday life, and family-level coping-remain relevant to understanding illness uncertainty in pediatric oncology. Future research should expand the sample scope, include healthcare professionals, and employ longitudinal or mixed methods designs to examine how families and healthcare systems jointly manage uncertainty over time.

## 5. Conclusions

This study highlights the multiple experiences of illness uncertainty among families of children with cancer and the strategies they develop to cope with it. Uncertainty arose from medical factors, such as delayed diagnosis and fluctuations in treatment, and was profoundly shaped by family and social contexts, including patterns of communication with healthcare professionals, disruptions to the structure of everyday life, financial pressure, and changes in caregiving roles. In response to ongoing uncertainty, families actively adopted a range of coping practices, including flexibly reorganizing family roles, assigning cultural meaning to the illness experience, strengthening emotional communication, and mobilizing external sources of support. These practices reflect both the resilience of families in the context of illness and their vulnerability under prolonged stress.

The findings suggest that healthcare systems should recognize uncertainty as a key experience that runs throughout the entire illness trajectory and provide appropriate psychosocial and structural support. Strengthening formal support systems, such as hospital social work services, and promoting coordination between peer support networks and community resources may help families manage uncertainty more effectively and reduce their long-term psychosocial burden. By adopting a family perspective, this study deepens understanding of uncertainty in pediatric oncology care and provides empirical evidence for the development of more responsive and family-centered models of care.

## Figures and Tables

**Table 1:** Sociodemographic profile of the sample.

Family No.	Participant Role	Educational Attainment/Occupation	Age (Years)	Sex of Child	Cancer Type	Treatment Duration Since Diagnosis (Days)	Time from Symptom Onset to Diagnosis (Days)	Sought Medical Care Outside Home Region	Place of Household Registration
F01	Child	Primary school	8	Female	Leukemia	242	50	Yes	Anhui
	Mother	Full-time caregiver	30						
	Father	Company employee	33						
F02	Child	Kindergarten	5	Male	Leukemia	125	95	Yes	Zhejiang
	Mother	Full-time caregiver	30						
	Father	Migrant worker	29						
F03	Child	Kindergarten	4	Female	Leukemia	150	40	Yes	Anhui
	Mother	Full-time caregiver	35						
	Father	Driver	44						
F04	Child	Middle School	13	Male	Leukemia	393	15	No	Shanghai
	Mother	Full-time caregiver	43						
	Father	Company employee	46						
F05	Child	Kindergarten	4	Male	Lymphoma	180	20	Yes	Guizhou
	Mother	Full-time caregiver	28						
F06	Child	Primary school	12	Male	Leukemia	213	180	Yes	Anhui
	Mother	Full-time caregiver	35						
	Father	Migrant worker	35						
F07	Child	Primary school	7	Female	Leukemia	125	18	Yes	Anhui
	Mother	Full-time caregiver	31						
	Father	Self-employed	35						
F08	Child	Primary school	9	Male	Leukemia	367	45	Yes	Fujian
	Mother	Full-time caregiver	35						
F09	Child	Primary school	10	Male	Leukemia	425	27	Yes	Sichuan
	Mother	Full-time caregiver	33						
F10	Child	Kindergarten	5	Male	Lymphoma	90	17	Yes	Fujian
	Mother	Teacher	36						
	Father	Newspaper editor	35						
F11	Child	Middle School	13	Female	Leukemia	307	10	No	Shanghai
	Mother	Company employee	40						
	Father	Company employee	42						
F12	Child	Primary school	8	Female	Leukemia	67	74	Yes	Jiangsu
	Mother	Full-time caregiver	29						

**Table 2:** Thematic analysis of data.

Themes	Subthemes	Examples of Participant Quotes
Sources of Uncertainty	The Unpredictable Illness Trajectory	*At first, my child was diagnosed with a cold. Later, when the doctors still could not figure out what was wrong, they even suspected it was a psychological problem related to school refusal. It was only after 95 days that my child was finally diagnosed with leukemia. During all that time, my child’s condition kept getting worse at home, and we became more and more afraid that the hospital and the doctors had delayed the diagnosis and treatment.* (F02f)
	Barriers in Communication with Healthcare Professionals	*The doctor said there was a strong suspicion of leukemia, but how serious it was could only be determined once treatment began. They also said that even medicine could not fully explain it. Hearing that was frightening. Before you even had time to process it, they moved straight on to discussing the next treatment plan.* (F07m)
	Disruptions to Everyday Life	*Sometimes I don’t even know what day of the week it is. I only remember how many days we’ve been in the hospital.* (F10f)
Navigating Uncertainty	Accepting Uncertainty	*There is an old saying that fortune and misfortune come together. So, I tell myself that after this illness, maybe greater blessings are still waiting for my child.* (F07f)
	Reorganizing Family Roles and Responsibilities	*This online job keeps me from constantly dwelling on my child’s illness. I feel less anxious, and earning money gives me a real sense of accomplishment. It feels like one of the few things I can still control.* (F07m)
	Intra-Family Communication and Mutual Support	*I did not expect that after our conversation, she really would begin to take eating seriously, which also reassured us a great deal.* (F01m)
	External Sources of Support	*My mother used all her retirement savings because she was afraid we would delay the child’s treatment if we ran out of money. She also kept trying to comfort me because she worried that I was under too much emotional strain.* (F08m)

## Data Availability

All data that support the findings of this study are available on request from the corresponding author. The data are not publicly available due to privacy or ethical restrictions.

## Supplementary Materials

The following supporting information can be downloaded at: https://www.mdpi.com/article/10.3390/healthcare14142127/s1, File S1: COREQ (COnsolidated criteria for REporting Qualitative research) Checklist; File S2: Semi-Structured Interview Guide.
